# P-636. Clinical Utility of Point-of-Care NAAT Compared to Antigen Testing for Influenza-Like Illness Outside of the Hospital

**DOI:** 10.1093/ofid/ofaf695.849

**Published:** 2026-01-11

**Authors:** Jordan Chase, Anita Mohandas, Annika Faucon

**Affiliations:** Cepheid, Somerville, MA; Cepheid, Somerville, MA; Truveta, Nashville, Tennessee

## Abstract

**Background:**

Various guidelines prefer molecular methods for diagnosis of influenza, SARS-CoV-2, and RSV due to limitations of antigen testing in sensitivity. 2024 ADLM guidelines specifically recommend NAAT for symptomatic outpatients when testing would impact patient management. Despite this, real-world data from 2021-2022 show that nearly half of symptomatic outpatients were tested using antigen. We aimed to assess the clinical utility of rapid NAAT testing for influenza-like illness in non-hospital outpatient compared to antigen.

**Table 1:**
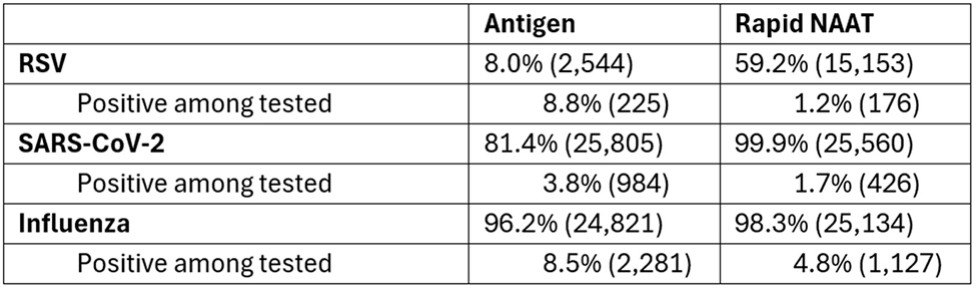
Total Testing and Positivity by Virus

**Table 2:**
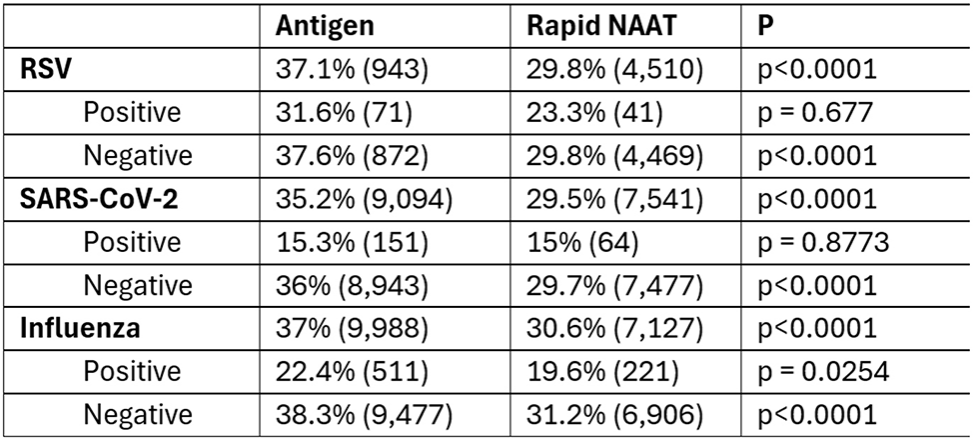
Antibiotic Use Within 14 Days, By Virus and Test Result

**Methods:**

Analysis was done on real-world EHR data in Truveta Studio. Patients were eligible if they had an antigen or rapid NAAT test for RSV, COVID-19, Flu A, and/or Flu B in non-hospital settings and were diagnosed with ILI from January 2023 – April 2025. Positivity rates and antibiotic use are reported in this unmatched analysis.

**Results:**

Among 57,281 patients tested outside hospitals, most had an antigen test (rapid NAAT = 25,582, 45%; antigen = 31,699, 55%). Most patients were tested for both SARS-CoV-2 and influenza; patients tested by rapid NAAT were more likely to be tested for RSV. Positivity rates by antigen testing were higher than rapid NAAT, which may reflect an imbalance in the sample population or result reporting. Patients receiving rapid NAAT testing had lower rates of antibiotic use at 14 days, regardless of virus tested for and whether the test result was positive.

**Conclusion:**

This analysis demonstrates that rapid molecular testing may result in better outcomes compared to antigen testing among patients evaluated in non-hospital settings. This supports existing recommendations by clarifying the clinical value of increased accuracy. Further research is warranted to assess additional outcomes, including use of additional testing, antiviral treatments, influenza-like illness-related healthcare encounters, as well as comparison of the existing cohort with propensity score matching or multivariate models to address the potential for confounders and biases.

**Disclosures:**

Jordan Chase, BA, Danaher: Stocks/Bonds (Public Company) Anita Mohandas, MS, Cepheid: Stocks/Bonds (Public Company) Annika Faucon, PhD, Truveta, Inc.: Advisor/Consultant|Truveta, Inc.: Employee

